# Assessing the risk factors before pregnancy of preterm births in Iran: a population-based case-control study

**DOI:** 10.1186/s12884-019-2183-0

**Published:** 2019-02-06

**Authors:** Maryam Soltani, Hamid Reza Tabatabaee, Shahin Saeidinejat, Marzieh Eslahi, Halime Yaghoobi, Ehsan Mazloumi, Abdolhalim Rajabi, Ali Ghasemi, Naeimeh Keyghobadi, Mostafa Enayatrad, Abed Noori, Seyyed Aliasghar Hashemi, Fatemeh Zolfizadeh, Sepideh Mahdavi, Tannaz Valadbeigi, Koorosh Etemad, Ali Taghipour, Cirruse Salehnasab, Mahmoud Hajipour

**Affiliations:** 1 Razi Clinical Research Development Unit(RCRDU), Birjand University of Medical Sciences(BUMS), Birjand, Iran; 2 Research Center for Health Sciences, Department of Epidemiology, School of Health, Shiraz University of Medical Sciences, Shiraz, Iran; 3 Department of Health Education and Health Promotion, School of Health Sciences, Mashhad University of Medical Sciences, Mashhad, Iran; 4 Department of Epidemiology, School of Health, Shiraz University of Medical Sciences, Shiraz, Iran; 5 Social Determinants in Health Promotion Research Center, Hormozgan University of Medical Sciences, Bandar Abbas, Iran; 6 Department of Epidemiology, School of Public Health, Hamadan University of Medical Sciences, Hamadan, Iran; 7 Department of Epidemiology, Faculty of Health, Iran University of Medical Sciences, Tehran, Iran; 8 Birjand University of Medical Sciences, Birjand, Iran; 9 Department of Biostatistics Epidemiology, Health Faculty, Shahid Sadoughi University of Medical Sciences, Yazd, Iran; 10 Epidemiology Department, School of Public Health, Shahid Beheshti University of Medical Sciences, Tehran, Iran; 11 Medical Education, Health Management and Social Development Research Center, Golestan University of Medical Sciences, Gorgan, Iran; 12 Health Care Management, Mother and Child Welfare Research Center, Hormozgan University of Medical Sciences, Bandar Abbas, Iran; 13 Clinical Research Development Unit, Imam Hossein Hospital, Shahroud University of Medical Sciences, Shahroud, Iran; 14 Department of Epidemiology, Environmental and Occupational Hazards Control Research Center, Faculty of Public Health, Shahid Beheshti University of Medical Sciences, Tehran, Iran; 15 Health Sciences Research Centre, Cancer Research Center, Department of Biostatistics and Epidemiology, School of Health, Faculty of Mashhad University of Medical Sciences, Mashhad, Iran; 16 Social Determinants of Health Research Center, Yasuj University of Medical Sciences, Yasuj, Iran; 17 Student Research Committee, Epidemiology Department, School of Public Health and Safety, Shahid Beheshti University of Medical Sciences, Tehran, Iran

**Keywords:** Preterm birth, Risk factor, Case-control, Iran

## Abstract

**Background:**

Preterm birth is a major cause of prenatal and postnatal mortality particularly in developing countries. This study investigated the maternal risk factors associated with the risk of preterm birth.

**Methods:**

A population-based case-control study was conducted in several provinces of Iran on 2463 mothers referred to health care centers. Appropriate descriptive and analytical statistical methods were used to evaluate the association between maternal risk factors and the risk of preterm birth. All tests were two-sided, and *P* values < 0.05 were considered to be statistically significant.

**Results:**

The mean gestational age was 31.5 ± 4.03 vs. 38.8 ± 1.06 weeks in the case and control groups, respectively. Multivariate regression analysis showed a statistically significant association between preterm birth and mother’s age and ethnicity. Women of Balooch ethnicity and age ≥ 35 years were significantly more likely to develop preterm birth (OR: 1.64; 95% CI: 1.01–-2.44 and OR: 9.72; 95% CI: 3.07–30.78, respectively). However, no statistically significant association was observed between preterm birth and mother’s place of residence, level of education, past history of cesarean section, and BMI.

**Conclusion:**

Despite technological advances in the health care system, preterm birth still remains a major concern for health officials. Providing appropriate perinatal health care services as well as raising the awareness of pregnant women, especially for high-risk groups, can reduce the proportion of preventable preterm births.

## Introduction

Preterm birth is defined as delivery before the gestational week 37 or day 259 [1–3]. It has been the most concerning complication among pregnant women and affects 10% of all pregnancies. Annually, 1 million neonatal deaths occur due to preterm birth [4]. It constitutes a large proportion of medical expenses and impose enormous economic burden on health care systems, families, and children [1]. Preterm birth is still a prevalent public health issue responsible for high perinatal mortality and long-term morbidity worldwide Despite improved perinatal care programs, it still remains a major leading cause of perinatal mortality, particularly in developing regions [5, 6].

Preterm birth is a multifactorial phenomenon, partially in association with immunologic, genetic, and environmental factors; however, its attributing factors have not yet been well studied [7, 8]. Previous studies concerned with preterm birth indicated that 45–50% of causes are unknown, 30% can be attributed to premature rupture of the membrane, and 15–20% are medical indications such as elective labor [8–10]. Recent studies have suggested that preterm birth is an independent risk factor for future cardiovascular diseases, cardiac ischemic diseases, and stroke [11, 12]. Due to the enormous economic and emotional burden of preterm birth and its associated complications, this study was conducted to examine the association between prenatal risk factors and preterm birth.

## Material and methods

**Fig. 1:**
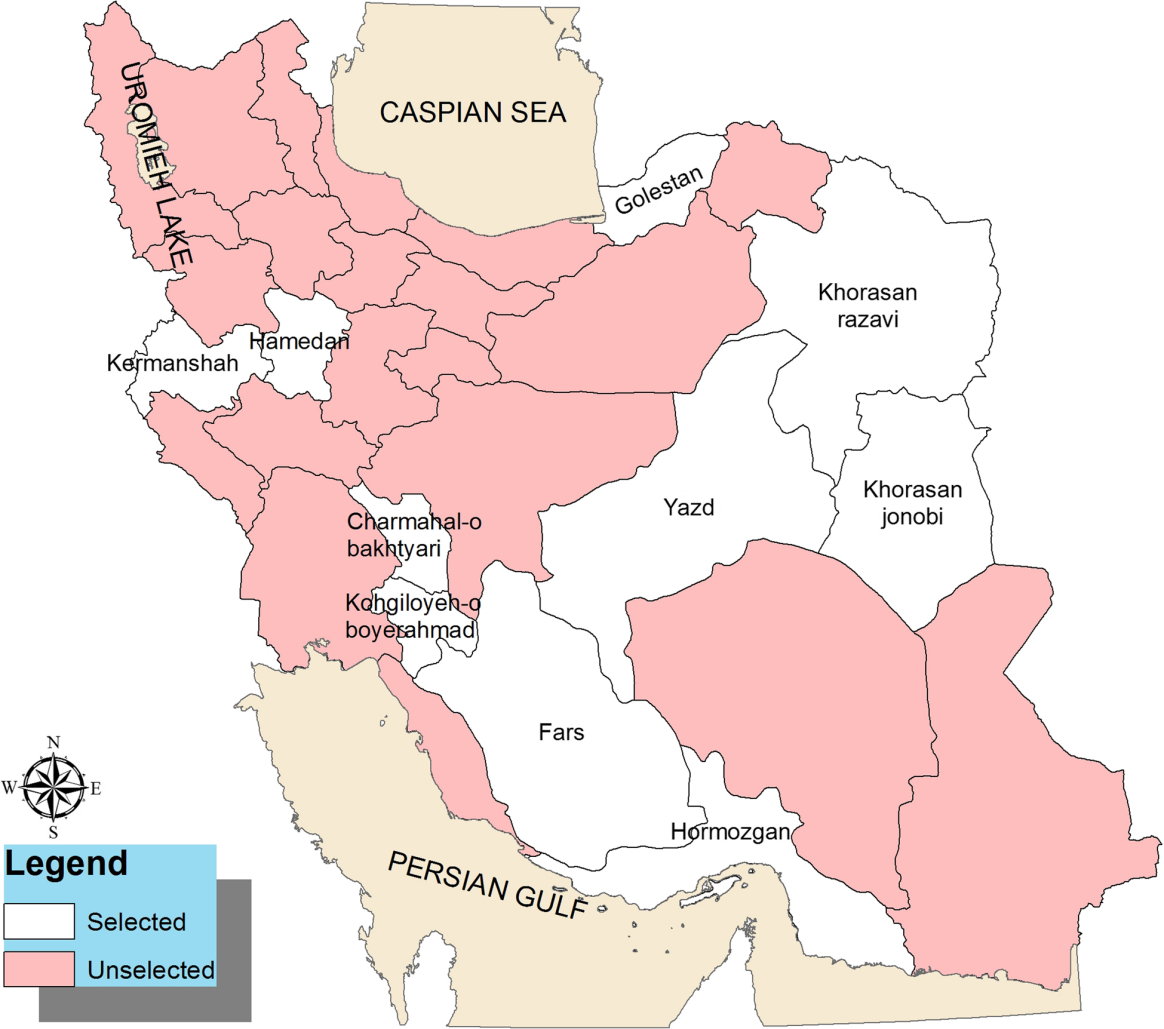
Study project locations in Iran. The population of the study was conducted on mothers that referred to a health care center in several provinces of Iran, including Fars, Hormozgan, Kermanshah, Hamadan, Kohgiloyeh, and Boyerahmad, Yazd, Southern Khorasan, Golestan, and city of Mashhad

This population-based case-control study was conducted on 2463 mothers, including 668 cases and 1795 controls, referred to a health care center in several provinces of Iran, namely, Fars, Hormozgan, Kermanshah, Hamadan, Kohgiloyeh, and Boyerahmad, Yazd, Southern Khorasan, Golestan, and city of Mashhad (Fig. 1). A rural health care center is a health facility in a village that provides health care for approximately 9000 people of that village and several neighboring villages. Health care providers at a rural health care center include a general physician and public health and midwifery experts. The rural health care center supervises and supports health care facilities in villages and is linked to its superior urban health care center. An urban health care center is a health facility in cities providing care to approximately 12,500 people. Health care providers, including a general physician and public health and midwifery experts, provide laboratory, pharmaceutical, radiological, and medical care in the urban health care centers. Experts at rural and urban health care centers register the provided health care to every family in their health records, such as health care for pregnant women. However, the registered information in the family’s health records were insufficient, and hence we collected additional data through interviews with the study participants.

The case group was defined as women who had preterm birth in a recent pregnancy, and the control group was defined as women who had full-term birth in a recent pregnancy [13–15]. The sample size ratio in the control and case groups was 3:1. Data were collected through interviews according to a check list containing demographic information (mother’s age, ethnicity, occupation and level of education, place of residence, and consanguineous marriage) and information on the previous pregnancies (the outcome of previous pregnancy, mode of delivery, and interpregnancy interval).

Study subjects were recruited through a multistage cluster sampling method. In the first stage regarding geographical divisions of Iran, nine clusters (provinces) were randomly selected. In the second stage, in each of the nine clusters (provinces), four clusters (cities) were randomly selected from the north, south, east, west, and central areas. In each city, two health care centers (one urban and one rural health care center) were randomly selected. In each health care center, 10 check lists were filled in by well-trained interviewers according to a protocol. In each center, data collection process was conducted simultaneously on the same day for cases and controls. Data of the control group were collected from a random sample of mothers referring to the health care center. If < 10 cases were available in each health care center, the remaining check lists were filled in the nearest center, and if there were > 10 cases, the check lists were filled in for a random sample of mothers. We tried to maintain the same size for the case and control groups.

According to literature review, considering mother’s age > 35 years as a risk factor (p_0_ = 0.3, p_1_ = 0.44, z_0.95_ = 2, z_(1-β)_ = 0.8, design effect = 2) [16] and using the proportion determination formula, the sample size was estimated as 370 for each study group. In this study, the association between preterm birth and 14 independent variables was evaluated. Therefore, taking into account an additional 20 samples for each independent variable, the total sample was calculated as 650 for each study group. The sample size was sufficient considering > 80% power of study.

### Sociodemographic variables

Sociodemographic information included mother’s age (age < 35 or ≥ 35 years), place of residence (urban vs. rural area), occupation (housewife/employee/cowhand/farmer/carpet weaver), level of education (illiterate/primary school/intermediate school/high school/academic gradation), ethnicity (Turk/Lor/Arab/Balooch/Torkaman/Fars/Kurd or others), and marriage (consanguineous vs. nonconsanguineous).

### Information on the previous pregnancies

This included history of abortion, stillbirth or cesarean section (yes/no), interpregnancy interval (the first pregnancy/< 1 year/1–3 years/> 3 years), BMI (normal/low weight/overweight/obese: grade 1, grade 2, and higher), and cycles of menstruation period (regular/irregular).

### The outcome variable

Preterm birth was the outcome variable, which was ascertained through questioning the exact gestational age at the time of birth.

### Statistical analysis

Descriptive statistical tests were performed for socio-demographic and pregnancy-related variables. Bivariate analysis was performed to identify the association of dependent and independent variables. Odds ratio was computed to see the strength of association between preterm birth and each of categorical variables. Adjusted odds ratio and their 95% confident interval were calculated by including all exposures with *p* value < 0.3 in the multivariate model to control for confounding effects [17]. Data were analyzed using SPSS version 19, with two-tailed tests at *p* ≤ 0.05 level of significance.

## Results

This study was conducted on 2463 mothers referred to health care centers (668 cases with a history of preterm birth and 1795 controls without a history of preterm birth). The mean gestational age at the time of birth was 31.5.” 4.03 vs. 38.8 ± 1.06 weeks for the case and control groups, respectively.

Our analysis revealed that 88.8% of cases and 94.0% of controls were 35 years of age. Regarding the ethnicity, 76.8% of cases and 64.1% of controls were Fars. Village dwellers comprised 51.9% of cases and 60.3% of controls. Regarding previous pregnancies, 5.8% of controls reported a history of stillbirth, 12.9% of cases and 11.4% of controls reported a history of cesarean section, and 15.3% of cases and 8.6% of controls had a history of abortion. Birth interval was longer than 3 years in 28.9% of cases and 30.4% of controls (Table 1).

**Table 1:** Demographic information and other characteristics of Mothers in cases and control groups (categorical variables)

Items	Preterm delivery		Total N (%)
	Control N (%)	Case N (%)	
All participants	1795 (73.0)	668 (27.0)	
Age(years)			
< 35	1674 (94.0)	588 (88.8)	2262 (92.6)
≥ 35	107 (6.0)	74 (11.2)	181 (7.4)
Level of Education			
Illiterate	76 (4.2)	39 (5.9)	115 (4.7)
Primary	366 (20.4)	172 (25.8)	538 (21.9)
Guidance	449 (25.0)	126 (18.9)	575 (23.4)
High school	689 (38.4)	251 (37.7)	940 (38.2)
Collegiate	213 (11.9)	78 (11.7)	291 (11.8)
Ethnic			
Tork	366 (21.7)	38 (6.1)	404 (17.5)
Lor	73 (4.3)	37 (5.9)	110 (4.8)
Fars	1083 (64.1)	479 (76.8)	1562 (67.5)
Kord	29 (1.7)	23 (3.7)	52 (2.2)
Arab	20 (1.2)	6 (1.0)	26 (1.1)
Balooch	11 (0.7)	6 (1.0)	17 (0.7)
Torkaman	99 (5.9)	32 (5.1)	131 (5.7)
Else	9 (0.5)	3 (0.5)	12 (0.5)
Occupation			
Housewife	1610 (90.9)	610 (91.9)	2220 (91.2)
Employee	110 (6.2)	40 (6.0)	150 (6.2)
Farmer& carpet weaver	31 (1.8)	7 (1.1)	38 (1.6)
Other	20 (1.1)	7 (1.1)	27 (1.1)
Place of Residence			
Urban	694 (39.7)	314 (48.1)	1008 (42.0)
Rural	1053 (60.3)	339 (51.9)	1392 (58.0)
Abortion history			
Yes	154 (8.6)	102 (15.3)	256 (10.4)
No	1641 (91.4)	566 (84.7)	2207 (89.6)
Stillbirth history			
Yes	104 (5.8)	122 (18.3)	226 (9.2)
No	1691 (94.2)	546 (81.7)	2237 (90.8)
Cesarean history			
Yes	205 (11.4)	86 (12.9)	291 (11.8)
No	1590 (88.6)	582 (87.1)	2172 (88.2)
Gap pregnancy(years)			
Upper than 3	537 (30.4)	189 (28.9)	726 (30.0)
Lower than 1	74 (4.2)	55 (8.4)	129 (5.3)
1–3	496 (28.1)	153 (23.4)	649 (26.8)
Primary pregnancy	661 (37.4)	257 (39.3)	918 (37.9)
Consanguineous marriage			
Yes	494 (28.1)	205 (31.3)	699 (29.0)
No	1263 (71.9)	450 (68.7)	1713 (71.0)
Supplements Consumption			
Yes, use regular	1449 (81.8)	535 (80.5)	1984 (81.4)
Yes, use not regular	213 (12.0)	91 (13.7)	304 (12.5)
Use not	109 (6.2)	39 (5.9)	148 (6.1)
BMI			
Normal	893 (53.6)	292 (48.7)	1185 (52.3)
Underweight	313 (18.8)	126 (21.0)	439 (19.4)
Overweight	329 (19.7)	121 (20.2)	450 (19.9)
Obesity grade1	101 (6.1)	55 (9.2)	156 (6.9)
Obesity grade 2	31 (1.9)	5 (0.8)	36 (1.6)
Regular Cycles of period			
Yes	1564 (90.2)	536 (82.7)	2100 (88.2)
No	170 (9.8)	112 (17.3)	282 (11.8)

Mothers with a consanguineous marriage were 1.32 times more likely to develop preterm birth (OR: 1.32; 95% CI: 1.04–1.67), those with a history of abortion were 1.57 times more likely to develop preterm birth (OR: 1.57; 95% CI: 1.08–2.27), and those with a history of stillbirth were approximately 4 times more likely to develop preterm birth (OR: 3.92; 95% CI: 2.76–5.57) (Table 2).

**Table 2:** Univariate logistic regression of risk factors for preterm delivery

Parameter	OR	95% CI	*P*- value
Place of Residence			
Rural	–	–	–
Urban	1.40	1.17–1.68	0.001
Level of Education			
Collegiate	–	–	–
Illiterate	1.40	0.88–2.23	0.155
Primary	1.28	0.93–1.76	0.122
Guidance	0.76	0.55–1.06	0.110
High school	0.99	0.73–1.33	0.973
Consanguineous marriage			
No	–	–	–
Yes	1.16	0.95–1.41	0.126
Abortion history			
No	–	–	–
Yes	1.92	1.46–2.51	0.001
Stillbirth history			
No	–	–	–
Yes	3.63	2.74–4.80	0.001
Cesarean history			
No	–	–	–
Yes	1.14	0.87–1.50	0.321
BMI			
Normal	–	–	–
Underweight	1.23	0.96–1.57	0.097
Overweight	1.12	0.87–1.44	0.350
Obesity grade1	1.66	1.16–2.37	0.005
Obesity grade 2	0.49	0.19–1.28	0.146
Age(years)			
< 35	–	–	–
≥ 35	1.96	1.44–2.68	0.001
Ethnic			
Tork	–	–	–
Lor	4.88	2.90–8.19	0.001
Fars	4.26	2.99–6.05	0.001
Kord	7.63	4.02–14.50	0.001
Arab	2.88	1.09–7.63	0.389
Balooch	5.24	1.84–15.0	0.001
Torkaman	3.11	1.85–5.23	0.001
Else	3.21	0.83–12.36	0.081
Regular Cycles of period			
Yes	–	–	–
No	1.92	1.48–2.48	0.001
Gap pregnancy(years)			
Upper than 3	–	–	–
Lower than 1	2.11	1.43–3.10	0.001
1–3	0.87	0.68–1.12	0.293
Primary pregnancy	1.10	0.88–1.37	0.374

Mothers aged 35 years or more compared to those younger than 35 years were 1.64 times more likely to develop preterm birth (OR: 1.64; 95% CI: 1.01–2.44). Regarding ethnicity, Balooch mothers compared to Turkish mothers were 9.27 times more likely to develop preterm birth (OR: 9.72; 95% CI: 3.07–30.78). Mothers with irregular cycles of menstruation period compared to those with regular cycles were 1.77 times more likely to develop preterm birth (OR: 1.77; 95% CI: 1.14–3.01). Regarding the interpregnancy interval, mothers with < 1-year interpregnancy interval compared to those with > 3 years were 1.85 times more liable to develop preterm birth (OR: 1.85; 95% CI: 1.14–3.01). However, no statistically significant association was observed regarding mother’s place of residence, level of education, supplement consumption, history of cesarean section, and BMI (Table 3).

**Table 3:** Multivariate logistic regression model of risk factors for preterm delivery

Parameter	OR	95% CI	*P*- value
Age(years)			
< 35	–	–	–
≥ 35	1.64	1.01–2.44	0.015
Ethnic			
Tork	–	–	–
Lor	4.55	2.50–8.29	0.001
Fars	4.07	2.72–6.09	0.001
Kord	5.80	2.74–12.28	0.001
Arab	1.71	0.50–5.80	0.484
Balooch	9.72	3.07–30.78	0.001
Torkaman	3.25	1.77–5.97	0.001
Else	3.69	0.85–16.09	0.108
Regular Cycles of period			
Yes	–	–	–
No	1.77	1.14–3.01	0.001
Gap pregnancy(years)			
Upper than 3	–	–	–
Lower than 1	1.85	1.14–3.01	0.012
1–3	0.81	0.60–1.11	0.198
Primary pregnancy	1.43	1.08–1.88	0.011
Consanguineous marriage			
No	–	–	–
Yes	1.32	1.04–1.67	0.019
Abortion history			
No	–	–	–
Yes	1.57	1.08–2.27	0.016
Stillbirth history			
No	–	–	–
Yes	3.92	2.76–5.57	0.001

## Discussion

The etiology of preterm birth has been a major concern in obstetrics worldwide. The cause of 50% of preterm births is unknown [18]. However, this study revealed a strong association between preterm birth and a history of abortion and stillbirth, ethnicity, interpregnancy interval, cycles of menstruation period, and consanguineous marriage. Consistent with other studies in this area, our study suggests an increased risk of preterm birth for mothers older than 35 years [18–22]. Martin et al. found an increased risk of preterm birth associated with older ages in women of high economic status [23]. The majority of studies indicated that the increased risk of preterm birth associated with increasing mother’s age may be confounded by socioeconomic factors or health complications associated with older ages, namely, hypertension, and renal diseases. Preventive strategies for older age mothers include providing appropriate health education and consultation, regular perinatal care during pregnancy, and encouraging mothers toward seeking effective family health [11, 22, 24]. Along with other studies, our study results suggest an association between preterm birth and ethnicity [4, 13, 15, 25]. Among all the studied ethnic groups, Balooch ethnicity was associated with an increased risk of preterm birth. This increased risk can be attributed to low socioeconomic status, high reproductive rates, low reproductive health status, insufficient reproductive knowledge, and poor nutrition. Preterm birth is affected by differences in ethnic groups regarding parents’ level of education, tobacco use, distress, and unfavorable experiences in life [13, 14, 26].

We observed an increased risk of preterm birth associated with consanguineous marriage, which was consistent with the results of other studies examining the genetic risk factors [27]. In addition, it should be considered that preterm birth can be affected by different environmental factors as well. Several studies suggested that even in the absence of genetic factors, preterm birth is associated with environmental factors such as socioeconomic status and tobacco use [27, 28]. Moreover, a number of studies suggested an interaction between preterm birth and parental genes or inheritance of human leukocyte antigen [29, 30].

Consistent with other studies, the present study demonstrated that a history of abortion and stillbirth is associated with an increased risk of preterm birth. Recent studies suggested an association between history of abortion and increased risk of preterm birth in subsequent pregnancies [11, 18, 31]. In addition, three large, population-based historical cohort studies and two large, case-control studies suggested that a history of abortion is a risk factor for preterm birth [31–33]. Several studies found an increased risk of preterm birth in association with more abortions and also indicated that various genetic and environmental factors can lead to repeated abortions [34–36]. It was also observed that abortion as the result of the last pregnancy is associated with an increased risk of preterm birth in gestational weeks < 32. The strength of this association decreases with increasing gestational weeks [31]. Although we observed an association between preterm birth and history of abortion, a population-based study in Pakistan reported conflicting results. Such inconsistent results can be attributed to the differences in the methods or limitations such as using data extracted from registry systems, lack of a control group, different definitions of gestational weeks for abortion, lack of control on potential biases, and confounding effects [31, 37].

We found that mothers with irregular cycles of menstruation period compared to those with regular cycles were more likely to develop preterm birth. Bonessen et al. also showed that regular cycles of menstruation period are associated with lower risk of prolonged pregnancy. In women with regular cycles of menstruation, the exact gestational age is clear and health care providers are not concerned with induction of pain [38].

Consistent with the results of other studies, our study results suggest an increased risk of preterm birth in mothers with < 1-year interpregnancy interval [39, 40]. Adams et al. suggested an increased risk of preterm birth associated with a 6- to 11-month interpregnancy interval. This risk decreased for interpregnancy intervals of > 47 months [41]. Krymko et al. [39] suggested that this association can be attributed to the presence of intrauterine infections before pregnancy or acute infections during pregnancy, mother’s physical weakness, emotional status, hormone secretion due to distress, or uterus contractions.

## Conclusion

Preterm birth is a multifactorial issue in obstetrics. Despite technological improvements in the health care system, it still remains a major concern for health officials. In the present study, ethnicity, history of abortion and stillbirth, irregular cycles of menstruation period, consanguineous marriage, and narrow interpregnancy intervals were found to be risk factors for preterm birth. Regarding preventive strategies, it is recommended that mothers be provided with reproductive health care before and during pregnancy, particularly in the high-risk groups, to reduce the proportion of preventable cases of preterm birth.

### Strengths and weaknesses of the study

The large sample size of the present study selected from several provinces includes different ethnic groups and socioeconomic status and increases the generalizability of results to the general population. In addition, data were collected by well-trained interviewers according to a predetermined protocol. However, the results of the present study should be interpreted with caution due to potentially uncontrolled confounding effects, recall bias related to history of abortion, and reporting bias due to self-report nature of data collection method. In addition, the time interval between the last abortion and current pregnancy was not recorded in the mother’s profile.

## Abbreviations

BMI: Body mass index
CI: Confidence interval
OR: Odds ratio
